# A comparative study of accuracy in major adaptive filters for motion artifact removal in sleep apnea tests

**DOI:** 10.1007/s11517-023-02979-9

**Published:** 2023-12-05

**Authors:** Yongrui Chen, Yurui Zheng, Sam Johnson, Richard Wiffen, Bin Yang

**Affiliations:** 1 Department of Physical, Mathematics and Engineering Sciences, University of Chester, Chester, CH1 4BJ UK; 2 Passion for Life Healthcare (UK) Ltd, Chester, CH1 2NP UK

**Keywords:** Sleep apnea, Motion artifact, Adaptive filter, SpO_2_, Heart rate

## Abstract

Sleep apnea is probably the most common respiratory disorder; respiration and blood oxygen saturation (SpO_2_) are major concerns in sleep apnea and are also the two main parameters checked by polysomnography (PSG, the gold standard for diagnosing sleep apnea). In this study, we used a simple, non-invasive monitoring system based on photoplethysmography (PPG) to continuously monitor SpO_2_ and heart rate (HR) for individuals at home. Various breathing experiments were conducted to investigate the relationship between SpO_2_, HR, and apnea under different conditions, where two techniques (empirical formula and customized formula) for calculating SpO_2_ and two methods (resting HR and instantaneous HR) for assessing HR were compared. Various adaptive filters were implemented to compare the effectiveness in removing motion artifacts (MAs) during the tests. This study fills the gap in the literature by comparing the performance of different adaptive filters on estimating SpO_2_ and HR during apnea. The results showed that up-down finger motion introduced more MA than left-right motion, and the errors in SpO_2_ estimation were increased as the frequency of movement was increased; due to the low sampling frequency features of these tests, the insertion of adaptive filter increased the noise in the data instead of eliminating the MA for SpO_2_ estimation; the normal least mean squares (NLMS) filter is more effective in removing MA in HR estimation than other filters.

**Graphical abstract:**

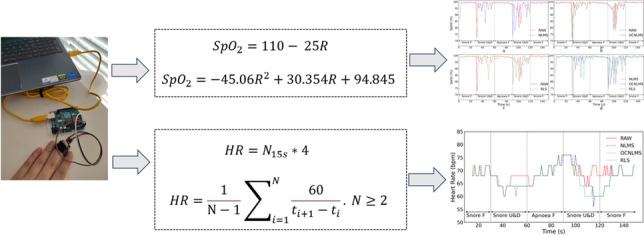

**Supplementary information:**

The online version contains supplementary material available at 10.1007/s11517-023-02979-9.

## Introduction

Sleep apnea is a sleep disorder characterized by a repeated cessation of airflow (apnea) or a lesser reduction in airflow (hypopnea) during sleep. The most common type of sleep apnea is obstructive sleep apnea (OSA). OSA is characterized by prolonged partial upper airway obstruction and/or intermittent complete obstruction that adversely affects ventilation during sleep and disrupts normal sleep patterns [1]. Each pause in OSA can last for at least 10 s to a few minutes and occur multiple times throughout the night, leading to a drop of 3% or more in blood oxygen levels and resulting in oxygen desaturation [2]. Statistics indicate that untreated OSA can have detrimental impacts on patients, including a higher risk of accidents with twice as many accidents per mile [3–5]. OSA is also associated with 1.9 times more cases of stroke, 3.9 times more cases of congestive heart failure [6, 7], 40% more cases of excessive daytime sleepiness [8, 9] and depression [10], a 30% increased risk of nocturnal cardiac arrhythmia [11], double the risk of occupational accidents [12], an eight times greater risk of COVID-19 in sleep apnea patients [13, 14], a 1.3 to 2.5 times increased risk of hypertension [15–17], and a 1.4 to 2.3 times increased risk of heart attack [9, 17].

The first comprehensive study to document the global prevalence of OSA was published by Benjafield et al. in 2019 [18]. It revealed that nearly 1 billion people globally suffer from this condition, with prevalence rates exceeding 50% in some nations. PSG is the gold standard procedure for OSA diagnosis. However, traditional PSG conducted in laboratory setting is overly complex, and only around 30 hospitals in the UK have the capability to perform PSG tests (information from Natus, PSG distributor in 2020). The existing home sleep apnea systems approved by the US Food and Drug Administration (FDA) are cumbersome, consisting of multiple parts and constructed from rigid materials that interface poorly with the skin [19, 20]. These drawbacks prompted the development of a low-cost, non-invasive, and convenient method to accurately diagnose sleep apnea, potentially within the familiar setting of one’s own home.

In 1938, Alrick Hertzman demonstrated the effectiveness of PPG as a non-invasive technique for measuring HR [21, 22]. Over the years, PPG devices have gained significant popularity in the healthcare system. They provide a rapid and non-invasive way to measure the volumetric variations of blood circulation using a light source and a photodetector placed on the skin. PPG sensors can detect changes in blood flow by measuring the intensity of reflected light from the tissue, as blood absorbs light more intensely than the surrounding tissues. Variations in blood volume are inversely proportional to the intensity of the reflected light. The PPG signal holds great promise as a sensor for detecting sleep apnea events because it captures crucial information about HR, respiration, and oxygen saturation.

SpO_2_, which stands for peripheral oxygen saturation, is an estimation of the oxygen saturation level, commonly measured using a PPG sensor. In individuals with a healthy respiratory condition, SpO_2_ values typically are in the range of 95% and 100%. A blood oxygen saturation level of less than 94% is considered hypoxic by the World Health Organization, while a level less than 90% may indicate the need for immediate medical action [23]. In the case of COVID-19 patients, who often experience significant respiratory failure around 10 days after initial infection, hypoxic SpO_2_ readings are a sign of hypoxia even in the absence of breathlessness [24].

PPG recordings can be impacted by various factors, with motion artifacts (MAs) being the primary source of signal modification. MA occurs when the PPG sensor shifts from its original position due to physical activity or body motion. This movement alters the path of light and subsequently affects the signals. Ambient light leaking into the gap between the PPG sensor surface and the skin is a common cause of MA. Additionally, changes in blood flow resulting from movements [25] can also be a contribution factor of MA. Several adaptive techniques have been applied to mitigate MA in PPG signals, including the least mean squares (LMS) method [26], normal least mean squares (NLMS) method [27], and recursive least squares (RLS) method [28]. The selection of an appropriate step size for an adaptive filter is also critical, which involves striking a balance between the adaptation speed and the steady-state noise. Various step sizes have been investigated to evaluate the quality of PPG signal by many researchers [29–31]. In contrast, the adaptive step-size (AS)-LMS algorithm provides both rapid convergence and minimal mean square error, as demonstrated by its high signal-noise ratio value [31]. Arunkumar and Bhaskar [32] developed a novel denoising algorithm that combined RLS, NLMS, and LMS adaptive filters. Their method demonstrated accurate HR estimation for the datasets involving activities such as running on a treadmill, arm recovery exercise, and fast arm movements.

While previous research has primarily focused on investigating the effectiveness of filters in mitigating MA, few studies have compared the accuracy of these filters. In this paper, we present a straightforward, non-invasive, continuous system for monitoring SpO_2_ and HR to mimic the sleep apnea scenarios at home. To the best of our knowledge, our system addresses this research gap by comparing the performance of different adaptive filters in estimating SpO_2_ and HR during apnea. Our study here encompasses the development of a non-invasive PPG monitoring system; evaluation of two measurement techniques for SpO_2_ and HR; assessing the relationship between SpO_2_, HR, and apnea; and finally the comparison of the signal impacts caused by different frequencies and directions of the artificial movements.

## Methods

### Human study

This study received ethical approval from the Research Ethics Committee of the Faculty of Science and Engineering at the University of Chester. All data were collected directly by the researcher herself, who was 33 years old, was 168-cm tall, weighted 70 kg, had a BMI of 24.8, and was a non-smoker and non-drinker. The researcher was in a stationary sitting position throughout the experiments and was in good overall health.

### Experiment design

According to a study conducted by Sally K et al. [33], the finger is considered the most suitable location for measuring HR, SpO_2_, and respiration rate while at rest. The experimental results of Zhang et al. [34] and Arghya Sur et al. [35] demonstrated that the middle finger exhibited a lower root-mean-square deviation (RMSE) for SpO_2_ error compared to the ring and index fingers. Therefore, for the data collection purposes of this study, a PPG sensor was placed on the left middle finger which was restricted to a fixed range of motion (using a vinyl tape with a 7.5-cm diameter). Finger movements can create a gap between the finger and the sensor, which allows red and infrared light from the sensor to scatter, introducing noise into the measurements. To minimize this noise, the experiment was conducted where the arm drove the finger to move while keeping the finger itself remains stationary; finger movements are used below to refer to this arm-driven movement.

**Fig. 1:**
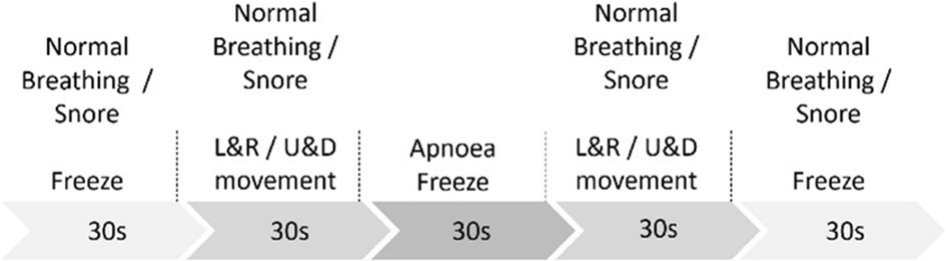
Schematic diagram of the experimental process. The breathing experiments were conducted in two scenarios: (1) normal breathing + apnea and (2) snore + apnea. Each breathing experiments consisted of five 30-s segments, and each segment involved a combination of finger movements and stillness. The finger movements were performed in different directions, including horizontal left and right directions, and vertical up and down directions. “L&R” represents left and right movements. “U&D” represents up and down movements

In order to investigate the relationship between SpO2 and apnea, breathing experiments were conducted in two scenarios: (1) normal breathing + apnea and (2) snore + apnea. Each breathing simulation consisted of five 30-s segments alternating between finger movement and stillness, as illustrated in Fig. 1. Once individuals entered the sleep state, only the routine body movements associated with breathing remained [36]. Therefore, the routine finger movements were performed in different directions and frequencies to assess the MA, including horizontal left and right directions, as well as vertical up and down directions, with motion frequencies of 0.5 Hz and 1 Hz, respectively. A total of eight sets of experiments were conducted, and they are (1) normal breathing with left and right movements at 0.5 Hz [NLR0.5], (2) normal breathing with left and right movement at 1 Hz [NLR1], (3) snoring with left and right movement at 0.5 Hz [SLR0.5], (4) snoring with left and right movement at 1 Hz [SLR1], (5) normal breathing with up and down movements at 0.5 Hz [NUD0.5], (6) normal breathing with up and down movements at 1 Hz [NUD1], (7) snoring with up and down movements at 0.5 Hz [SUD0.5], and (8) snoring with up and down movements at 1 Hz [SUD1]). Each set of experiments was repeated 10 times, lasting for 2.5 min, with a 10-min break between each experiment to allow for breathing recovery before proceeding to the next set.

Breathing experiments were conducted to simulate the respiratory patterns during sleep. For the normal breathing

simulation, the inhalation and exhalation cycles were set at 3 s each. Conversely, the simulated snoring experiment involved longer inhalation and exhalation cycles, with each cycle lasting 5 s.

### Hardware

**Fig. 2:**
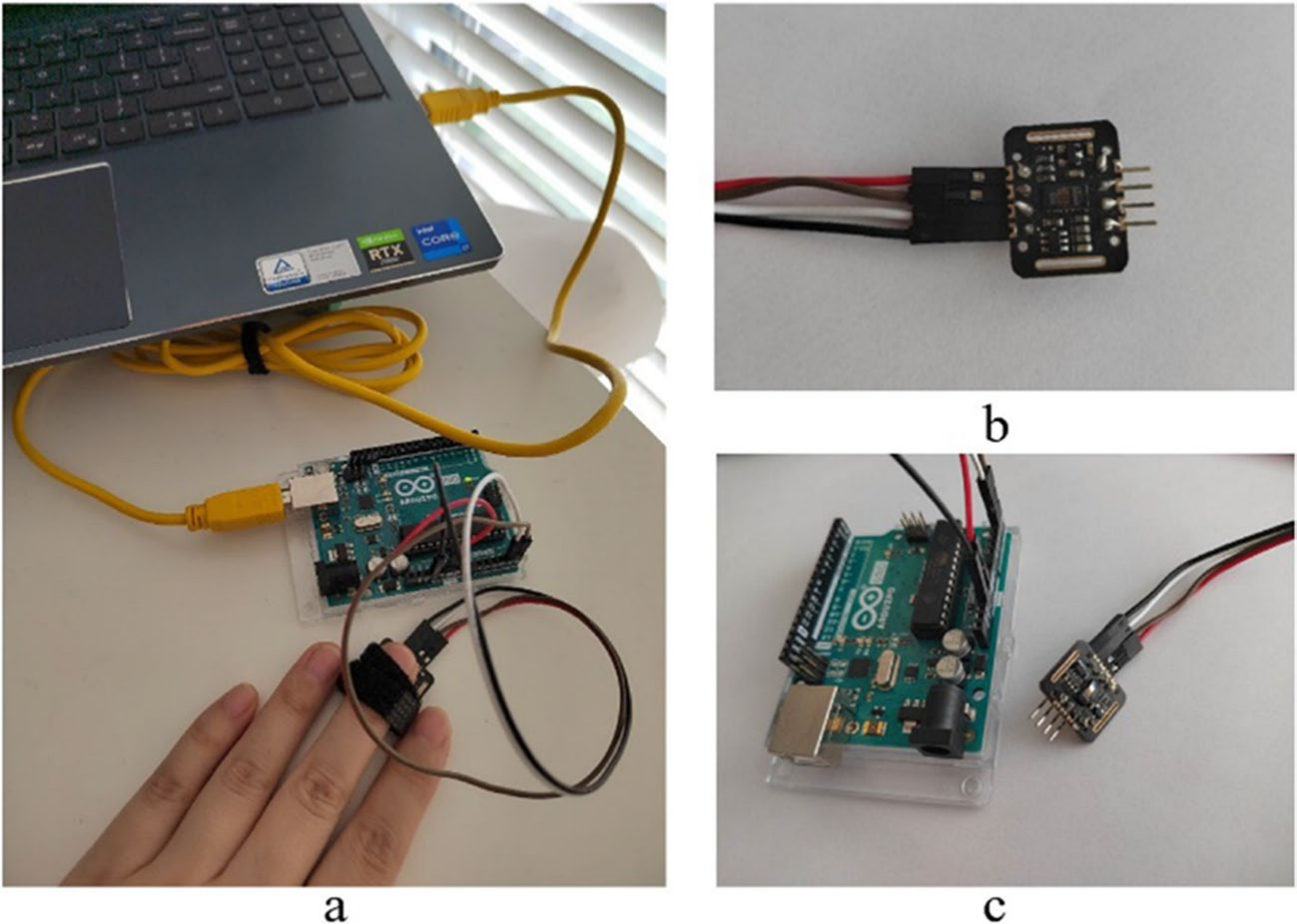
**a** PPG monitoring system. **b** PPG sensor MAX30102. **c** MAX30102 connect with Arduino board

The MAX30102 digital PPG chip, developed by Maxim Integrated (San Jose, CA, USA), was used as the PPG sensor in this study. The MAX30102 sensor comprises red (peak wavelength 660 nm) and infrared (peak wavelength 880 nm) light-emitting diodes and a photodiode to measure the reflected light. The MAX30102 sensor includes an analogue to digital converter (ADC) and an *I*^2^C interface integrated into the sensor itself to minimize noise and artifacts created between the photodiode and the ADC. Figure 2 depicts the PPG monitoring system, the MAX30102 sensor, and the connection of the sensor to the Arduino board.

Considering factors such as power efficiency, cost-effectiveness, and the sufficiency of data for analysis, the data acquisition system was used to capture and digitize the analogue PPG signal at a rate of 25 samples per second [37–41]. The PPG signal consists of pulsatile (AC) and non-pulsatile (DC) components [42], as shown in Supplementary Fig.1. The signal primarily consists of a static (DC) component, which captures light unaffected by the pulsatile variations in arteries. This DC component is chiefly influenced by ambient light, direct light interference between the LED and photodiode (PD), and light reflections from tissues, venous blood, and non-pulsating arterial blood. A relatively smaller portion of the signal constitutes an alternating component (AC), arising from the pulsations within the arterial bed. The AC and DC components were obtained and analyzed using Python code.

### SpO_2_ extraction and estimation

To calculate SpO_2_, both an empirical formula and a customized formula provided in the Maxim Integrated^TM^ sample code [33] were used. The “findpeaks” function was initially applied to locate the actual peaks and valleys, allowing determination of the AC and DC value for both the red and infrared channels. The function can find all local maxima by simple comparison of neighboring values. The AC and DC component of the pulsative waveform were stored as a mean of two consecutive peaks/valleys in integer variables (AC_Red_, AC_IR_, DC_Red_, DC_IR_), which maximized retention of original data while reducing errors. A ratio (R) of the AC and DC components of the red signal (Red), divided by the ratio of the AC and DC components of the infrared (IR) signal was then calculated (1) [43]. Subsequently, SpO_2_ was calculated using both the empirical formula (2) [44–47] and Maxim customized formula (3) [33, 42, 48]:

1 $$R=\frac{A{C}_{\mathrm{Red}} / D{C}_{\mathrm{Red}}}{ A{C}_{\mathrm{IR}} / D{C}_{\mathrm{IR}}}$$

2 $${SpO}_{2}=110- 25R$$

3 $${SpO}_{2}=-45.06{R}^{2}+30.354R+94.845$$

Since SpO_2_ levels below 70% are rarely observed, even in patients with severe OSA, a range of 0*<*R*<*1.2 was used to exclude abnormal R results, to ensure the calculated value in a range of 66% *<* SpO_2_ *<* 100% [49, 50].

### HR extraction and estimation

The AC component of the PPG signal is generated by the cardiac synchronous variations in blood volume that arise from heartbeats. After determining the peak and valley locations, the time difference between two consecutive peaks was obtained to calculate the average value of HR using (4). This provides the instantaneous HR.

4 $$HR=\frac1{\mathrm N-1}\sum\nolimits_{i=1}^N\frac{60}{t_{i+1}-t_i}.\;N\geq2$$

Another method for calculating HR involves counting the number of peaks within each 15-s window (as each apnea can last for at least 10 s [2]) and then multiply this number by 4 to obtain the beats per minute. This HR is referred to as resting HR and is frequently used by nurses and doctors to quickly check a patient’s pulse. It can also be used for routine self-examination.

However, obtaining an accurate estimation of the HR is not straightforward since PPG signals are vulnerable to noise, which can significantly interfere with HR calculation. To address this issue, we applied a band-pass filter with a frequency range of 0.5–3 Hz to remove low and high noise from the signal, accounting for both low and high HR (30–180 bpm).

### MA removal

Adaptive filters are commonly used to mitigate noise interference in PPG signals. Many types of adaptive filters are available, including affine projection (AP), generalized maximum correntropy criterion (GMCC), generalized normalized gradient descent (GNGD), least Lncosh (Llncosh), least-mean-fourth (LMF), least-mean-square (LMS), normalized least-mean-fourth (NLMF), normalized least-mean-square (NLMS), normalized sign-sign least-mean-square (NSSLMS), online centered normalized least-mean-square (OCNLMS), recursive least squares (RLS), sign-sign least-mean-square (SSLMS), variable step-size least-mean-square (VSLMS) with Ang’s adaptation, variable step-size least-mean-square (VSLMS) with Benveniste’s adaptation, and variable step-size least-mean-square (VSLMS) with Mathews’s adaptation. A detailed comparison of these filters can be found in the “Supplementary information” section. After comparing the different filters, it was found that the NLMS, RLS, and OCNLMS filters were the most effective.

The selected adaptive filters were applied to the raw PPG signal as a preliminary step to calculate the SpO_2_ and HR, while the band-pass filter was used to calculate the HR. It should be noted that no band-pass filter was employed in measuring SpO_2_, as its inclusion resulted in a significant drift in the SpO_2_ values.

## Experiment and results

### SpO_2_

#### Empirical formula vs customized formula

**Fig. 3:**
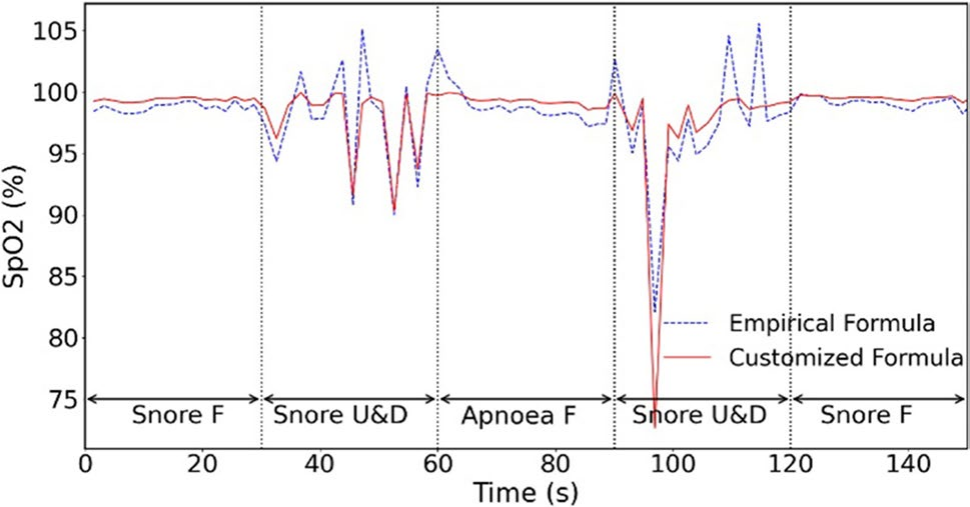
Comparison of SpO_2_ values calculated by empirical (dashed line) and customized formulas (solid line). Note “F” in the figure represents “freeze” and indicates a stationary hand. “U&D” represents up and down movements

In Fig. 3, the results obtained from two different formulas for calculating SpO_2_ are compared. Notably, the empirical formula demonstrates significant errors, as the calculated SpO_2_ levels exceed 100%. In contrast, the customized formula produces relatively accurate SpO_2_ values. Furthermore, the SpO_2_ values derived from the empirical method tend to be lower than those calculated using the customized formula.

During the first minute of the experiment, the researcher was instructed to breathe normally and only move their finger, resulting in expected blood oxygen values between 95 and 100% as mentioned previously. Any values below 95% is considered abnormal. Table 1 shows the number of abnormal values observed during the first minute for both empirical and customized formulas. Additionally, a statistical comparison was conducted for the abnormal values where SpO2>100% throughout the entire experiment.

**Table 1:** Error rates for both empirical and customized formulas

Error rate (%)	Empirical formula		Customized formula	
	SpO_2_ < 95%	SpO_2_ > 100%	SpO_2_ < 95%	SpO_2_ > 100%
NLR 0.5	2.5	27.1	1.9	0
NLR 1	1.2	9.3	0.5	0
NUD 0.5	2.1	15.9	1.6	0
NUD 1	0.9	19.7	0.3	0
SLR0.5	2.4	26.5	2.2	0
SLR 1	3.7	13.9	2.7	0
SUD 0.5	2.6	15.7	2.2	0
SUD 1	5.3	17.9	3.5	0
Mean	2.59	18.18	1.87	0

**Fig. 4:**
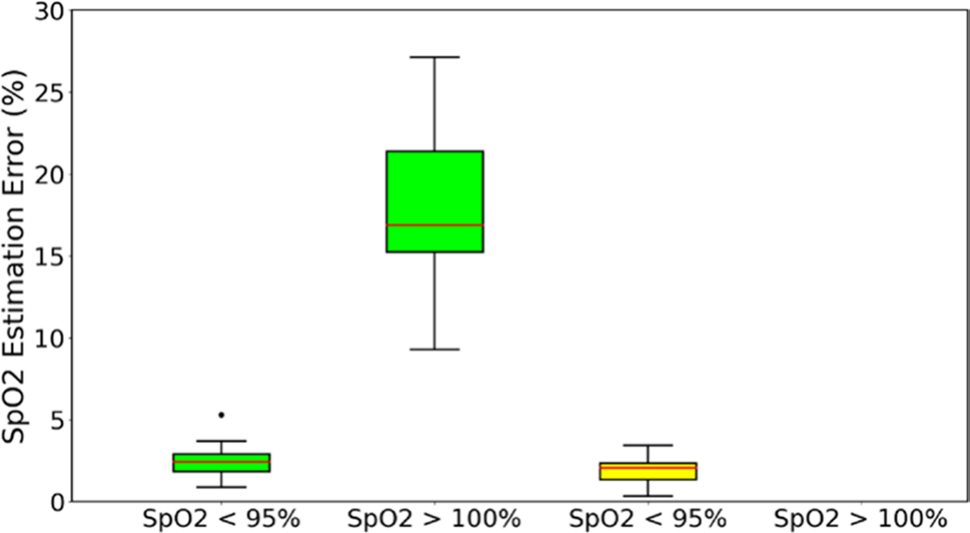
Comparison of the error rates for SpO_2_ < 95% during first minute and SpO_2_ > 100% throughout the experiment for both empirical (green box) and customized (yellow box) formulas. Results show that the customized formula has less error in estimating SpO_2_ than empirical formula

Our study found that the customized formula performed better than the empirical formula in estimating SpO_2_ values, particularly by avoiding any errors for values above 100% throughout the entire experiment. The box plots in Fig. 4 demonstrated that the empirical formula had a significantly higher error rate (18.18%) compared to the customized formula, which had an error rate of 0%.

Moreover, during the first minute of the experiment, the customized formula had superior performance compared to the empirical formula, with an error rate of 1.87% for SpO_2_ values less than 95%. This error rate was only slightly lower than the empirical formula’s error rate of 2.59%. Based on these findings, we have selected the customized formula as the preferred method for estimating SpO_2_ values.

#### Adaptive filter comparison

**Fig. 5:**
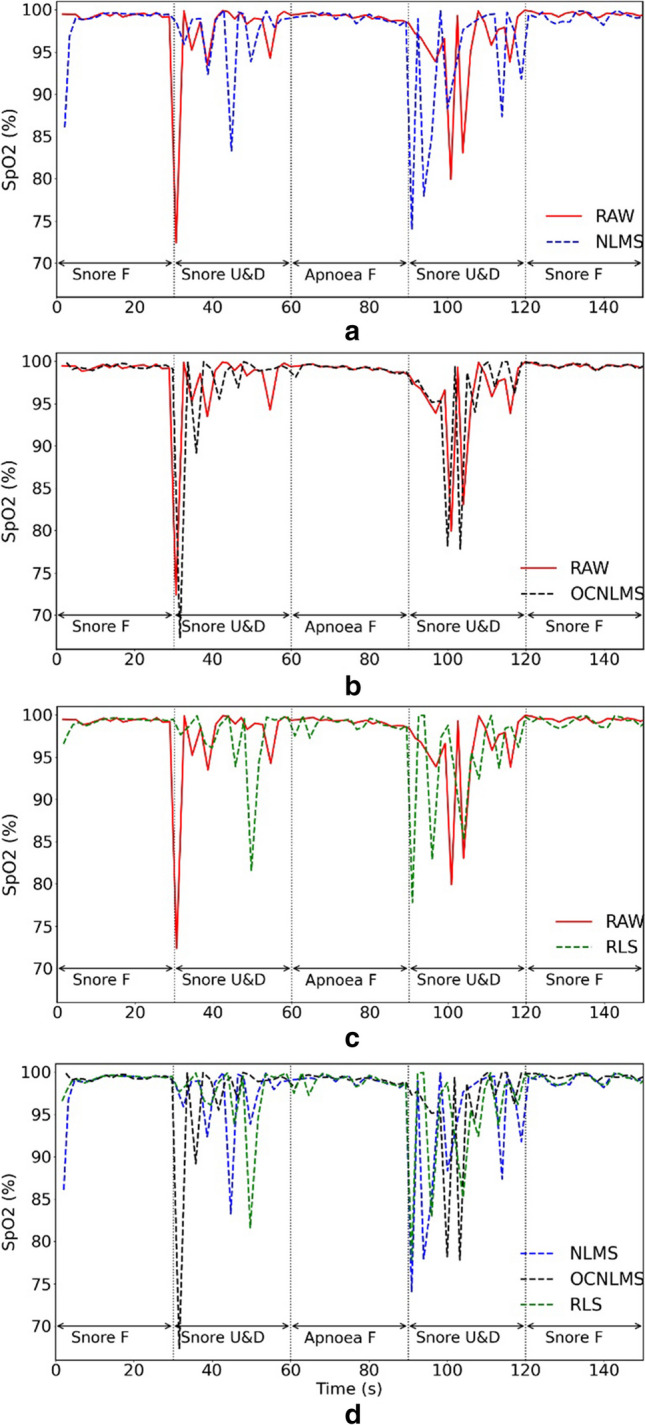
Comparison of the performance of raw data (RAW) and data processed with NLMS, OCNLMS, and RLS adaptive filters on blood oxygen saturation during snore experiment with 1-Hz up and down movements. **a** RAW vs. NLMS. **b** RAW vs. OCNLMS. **c** RAW vs. RLS. **d** NLMS vs. OCNLMS vs. RLS. Note “F” in the figure represents “freeze” and indicates a stationary hand; “U&D” represents up and down movements

Fig. 5 illustrates the SpO_2_ results of the snore experiment involving vertical up and down movements at a frequency of 1 Hz. The x-axis represents the time in seconds, while the y-axis represents blood oxygen saturation percentage. The red solid line represents the raw data without any added filters, while the other dashed lines represent the raw data with different filters applied. NLMS, OCNLMS, and RLS represent the test raw data (RAW) with NLMS, OCNLMS, and RLS filters applied, respectively.

The waveform appears consistent and smooth during the freeze finger phase but exhibits variation during the motion phase, which verifies that movement has an impact on SpO_2_ values. The lines and values also show variation with the addition of different adaptive filters. The line remains stable in the part where the fingers were stationary. However, the raw values in the range of 40–50 s, once the filter was applied, moved outside of 98–100% range. Apnea can lead to a drop in SpO_2_ levels, but the SpO_2_ level is maintained at high levels for healthy individuals, even after a few minutes of breath holding [50, 51]. Therefore, for the researcher, a 30-s breath holding (mimicking apnea) will not result in a SpO_2_ drop below 90%. Table 2 presents a comparison of the error rates between the raw data and data processed with the NLMS, OCNLMS, and RLS filters for SpO_2_ values less than 90% throughout the experiment.

**Table 2:** Error rates for raw data and data with different adaptive filters

Error rate (%)	SpO_2_ < 90% RAW	SpO_2_ < 90% NLMS	SpO_2_ < 90% OCNLMS	SpO_2_ < 90% RLS
NLR 0.5	1.93	2.64	1.84	2.61
NLR 1	2.12	3.83	2.14	3.06
NUD 0.5	2.66	4.26	3.86	4.19
NUD 1	2.83	5.95	3.41	3.63
SLR0.5	1.74	3.91	2.2	3.65
SLR 1	1.19	1.97	1.74	1.75
SUD 0.5	2.4	3.27	2.63	2.3
SUD 1	3.46	4.15	4.11	3.98
Mean	2.28	3.74	2.72	3.14

We observed that, regardless of whether an adaptive filter is used or not, the quality of most signals deteriorates as movement frequency increases, which causes an increase in errors of SpO_2_ estimation.

With respect to the movement direction, the vertical up and down movements demonstrated significantly higher error rates in the blood oxygen test compared to horizontal left and right movements. This indicates that the up-down motion introduced more MA than the left-right motion.

Regarding the total error rate, the raw data without the adaptive filter exhibited the lowest error rate at 2.28% for SpO_2_*<*90% throughout the experiment, as depicted in the box plots. Comparing the data with the filters, the OCNLMS filter had the best result (2.72%), followed by the RLS filter (3.14%) and the NLMS filter (3.74%).

**Fig. 6:**
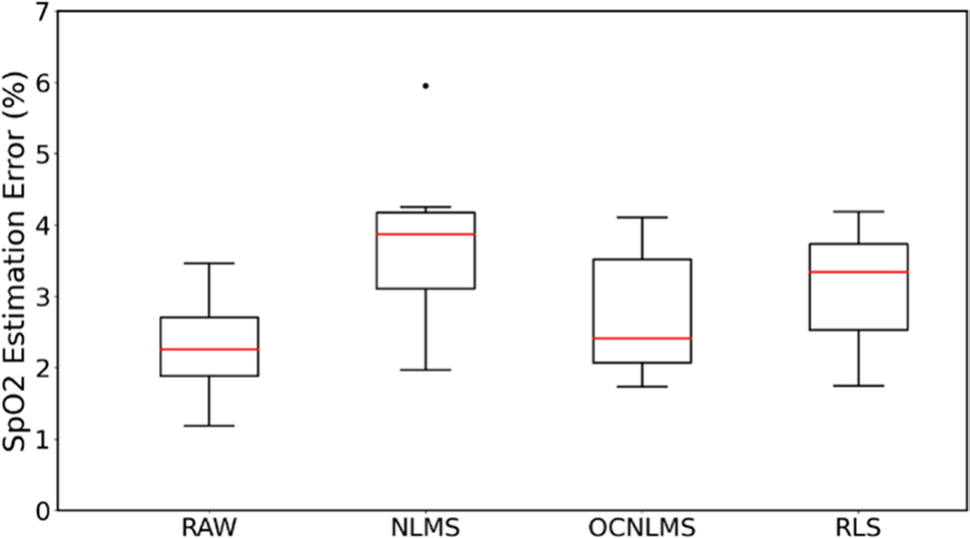
Comparison of the error rates between raw data (RAW) and data processed with different adaptive filters for SpO_2_<90% throughout the experiment: RAW vs. NLMS vs. OCNLMS vs. RLS. Results indicate data with OCNLMS filter has fewer errors than NLMS and RLS filters

**Fig. 7:**
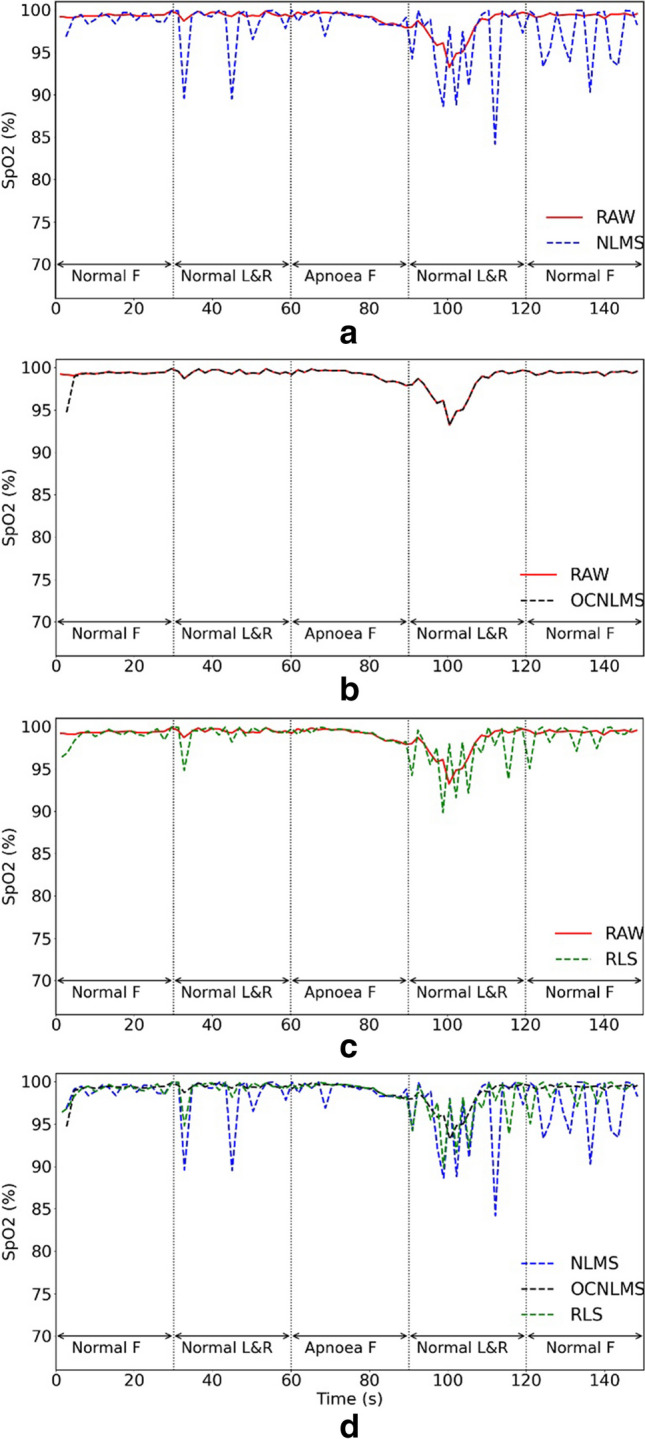
Comparison of raw data (RAW) vs data with NLMS, OCNLMS, and RLS adaptive filters on SpO_2_ during normal breathing experiment with 0.5-Hz left and right movements. **a** RAW vs. NLMS. **b** RAW vs. OCNLMS. **c** RAW vs. RLS. **d** NLMS vs. OCNLMS vs. RLS. Note “F” in the figure represents “freeze” and indicates a stationary hand. “L&R” represents left and right movements

The inclusion of the adaptive filter seems to amplify noise in the data rather than mitigating motion artifacts, as evident from Figs. 6 and 7 portrays an experiment characterized by minimal motion artifacts and clean data; however, the situation alters when the filter is introduced.

The red solid line in Fig. 7 represents the raw SpO_2_ data, which oscillate around 99% for the first 75 s. However, after 15 s of apnea event occurrence, the waveform starts to decline, and the SpO_2_ values continue to drop even after breathing resumes. It takes approximately 20 s for the SpO_2_ values to return to 99% after breathing resumes. Following this, the values are still fluctuated slightly compared to the initial finger freeze section, but the fluctuations remain within 1% and can be considered stable. This observation suggests a delay between the onset of apnea and subsequent decrease in SpO_2_ levels. According to a study by Chang et al. [52], the average delay time between an apnea (hypopnea) event and a 3% drop of SpO_2_ was found to be 19.3±9.6s. The result of our experiment is consistent with the conclusion drawn by Chang et al. The data processed with the OCNLMS filter did not change significantly, while the data processed with the NLMS and RLS filters were exactly the opposite, with the lines drastically fluctuating during the finger movement sections, even in the last 30 s.

Boxplot is a graphical representation of a dataset’s distribution; it provides a visual summary of the data’s central tendency, spread, and any potential outliers [53]. The box in the plot represents the interquartile range (IQR), which encompasses the middle 50% of the data. The whiskers extend from the edges of the box to the minimum and maximum values within a specified range. Any data points outside this range are considered outliers.

Table 3 provides a comparison of the percentage of data within the whiskers for both the raw data and the data processed with filters. The results indicate that OCNLMS and RLS filters have a slightly larger percentage of data within the whiskers compared to the raw data (87.89%), with percentages of 87.92% and 88.46%, respectively. NLMS, on the other hand, has a slightly smaller percentage of data within the whiskers, with a percentage of 87.43%.

**Table 3:** Percentage of data within whiskers for raw data and data with different adaptive filters

Percentage of data within whiskers (%)	RAW	NLMS	OCNLMS	RLS
NLR 0.5	86.87	87.2	88.27	88.67
NLR 1	90.71	89.43	91.13	90.33
NUD 0.5	86.94	86.34	87.67	86.84
NUD 1	87.95	88.53	87.31	88.12
SLR0.5	86.95	87.94	87.8	88.26
SLR 1	88.86	86.82	88.97	91.39
SUD 0.5	85.61	84.78	85.05	85.83
SUD 1	89.26	88.42	87.14	88.24
Mean	87.89	87.43	87.92	88.46

These percentages within the whiskers are important because they give an indication of how tightly the data is clustered within IQR. A larger percentage within the whiskers suggests that the data is more concentrated within the IQR and less outliers. In this case, the differences between the filter types and the raw data are relatively small, suggesting that the filters have a subtle effect on the MA removal.

It is worth highlighting that the approach taken in this study for removing motion artifacts from SpO_2_ estimation relied solely on the use of an adaptive filter, in contrast to other studies [27, 31, 32] that utilize multiple filters. Consequently, it becomes challenging to definitively determine which specific filter contributed to the improvement in results. While this experiment was designed for a direct and clear comparison of different filters, the performance of the adaptive filter did not meet expectations. In certain cases, it introduced additional noise to the data, thereby interfering with the analysis. In previous studies [51, 54], where the sampling frequency ranged from 200 to 400 Hz, and the adaptive filter would effectively smooth out the lines. However, the sampling frequency for our experiment was only 25 Hz, which resulted in less satisfactory results compared with higher sampling frequencies.

### HR

#### Resting HR vs instantaneous HR

**Fig. 8:**
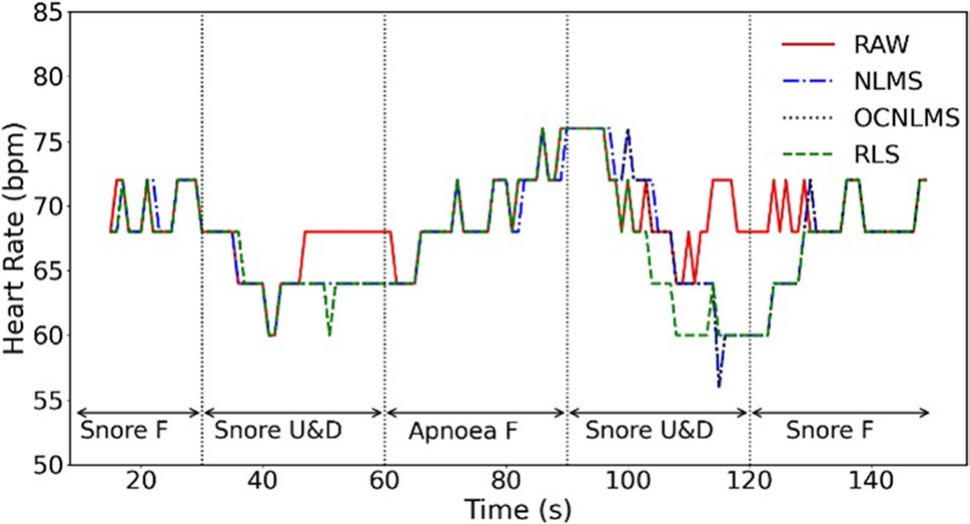
Comparison of raw data (RAW) vs data processed with NLMS, OCNLMS, and RLS adaptive filters on resting HR during snore experiment with 1-Hz up and down movement. Note “F” in the figure represents “freeze” and indicates a stationary hand. “U&D” represents up and down movements

**Fig. 9:**
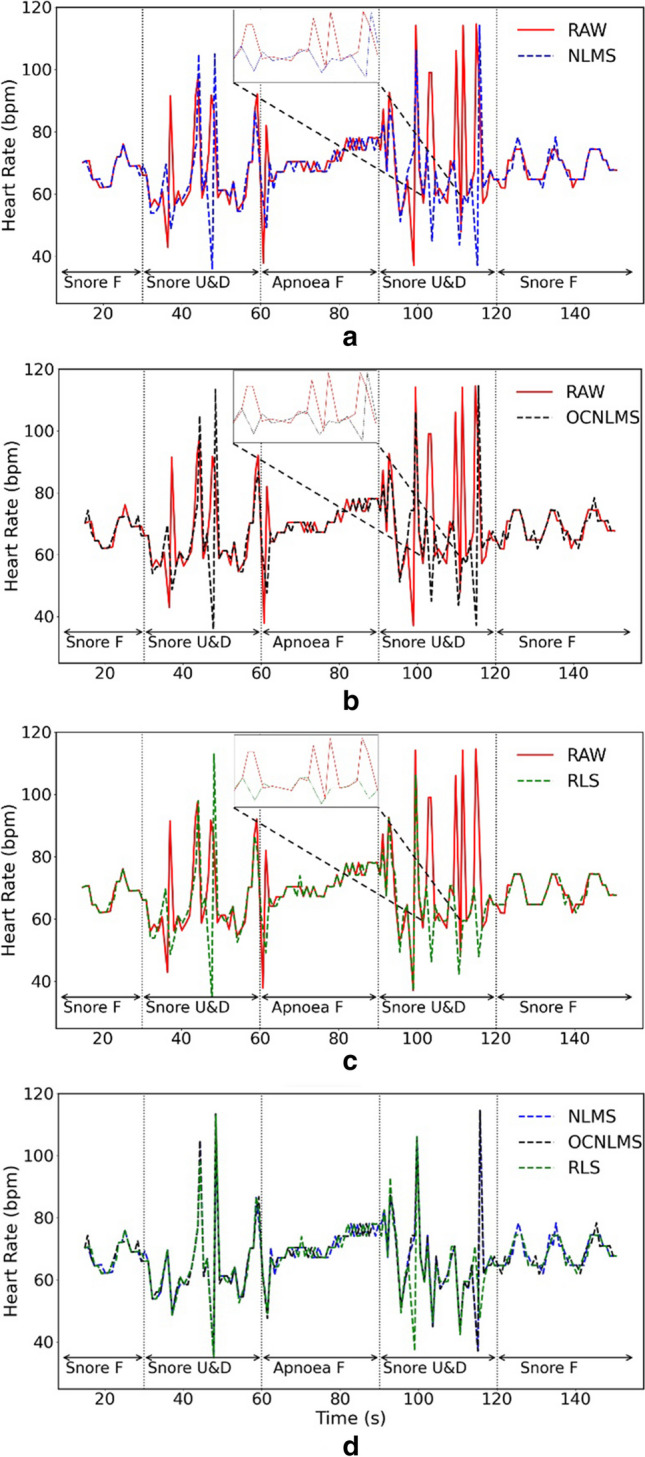
Comparison of raw data (RAW) vs data with NLMS, OCNLMS, and RLS adaptive filters on instantaneous HR during snore experiment with 1-Hz up and down movements. **a** RAW vs. NLMS. **b** RAW vs. OCNLMS. **c** RAW vs. RLS. **d** NLMS vs. OCNLMS vs. RLS. Note “F” in the figure represents “freeze” and indicates a stationary hand. “U&D” represents up and down movements. The time between 102 and 116 s in a, b, and c was magnified to provide a detailed illustration of the adaptive filter's effect on MA, demonstrating its effective capability to flatten the waveform

**Fig. 10:**
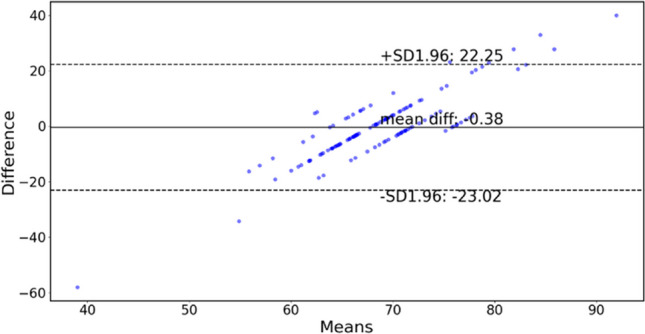
Comparison of resting HR and instantaneous HR using Bland-Altman plot during snore experiment with 1-Hz up and down movements. Illustrates large discrepancy between the resting HR and the instantaneous HR

Figures 8 and 9 display the results of two methods for determining HR in an experiment that involved snoring with up and down movements at a frequency of 1 Hz. Figure 10 depicts the Bland-Altman analysis of the experiment, a mean bias of 0.38 bpm was obtained, with the upper and lower limit of agreement (LOA) bounds being 22.25 and −23.02 bpm, respectively, at a 95% confidence interval. The results indicate a lack of agreement between the two approaches. Furthermore, Fig. 9 demonstrates that the estimated instantaneous HR varies significantly during finger movement, but remains stable during resting HR. This suggests that MA have a greater impact on the instantaneous HR than the resting HR. Therefore, resting HR was selected to estimate the HR value.

Furthermore, as observed in Fig. 8, the HR maintains in a steady fluctuation throughout the experiment. It is worth noting that when breathing resumes after an apnea period, there is a significant rise in HR. This phenomenon commonly occurred during sleep and is known as an involuntary reflex [55]. When a person stops breathing during sleep, the longer the oxygen deprivation, the more likely the HR tends to decrease. Subsequently, involuntary reflexes cause the person to wake up at the end of breathing cessation period, resulting in a rapid increase in HR. Although the experiment was performed while awake, a similar involuntary reflex occurred during the apnea period where the HR increased to obtain more oxygen. A significant rise in HR was observed when the breathing resumes, as a large amount of oxygen was obtained. These results have been verified in the rest of the experiments. It can be concluded that prolonged apnea results in an increase in HR to obtain more oxygen, which gradually decreases and stabilizes upon resumption of normal breathing.

#### Adaptive filter comparison

**Fig. 11:**
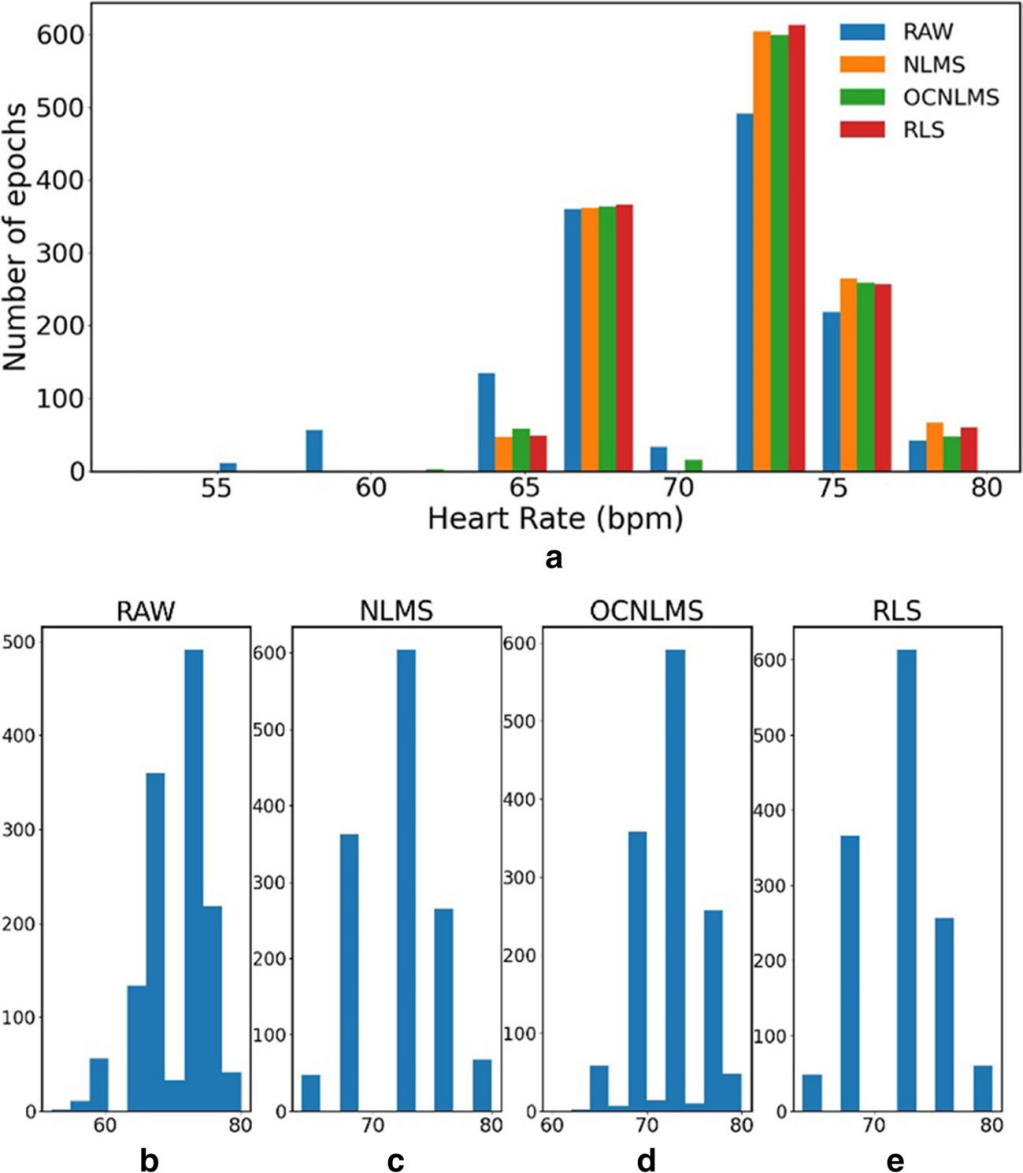
Histogram of resting HR of raw data (RAW) and data with NLMS, OCNLMS and RLS filter on snore experiment with 0.5Hz up & down movement. a. RAW vs. NLMS vs. OCNLMS vs. RLS b. RAW c. NLMS d. OCNLMS e. RLS

Fig. 11 shows the distribution histogram of snore experiment with up and down movements at a frequency of 0.5 Hz. The raw data along with data processed using NLMS, OCNLMS, and RLS filters are overlaid, and there are a significant number of outliers in the range of 50–60 bpm, whereas the filters effectively filter out these outliers.

**Fig. 12:**
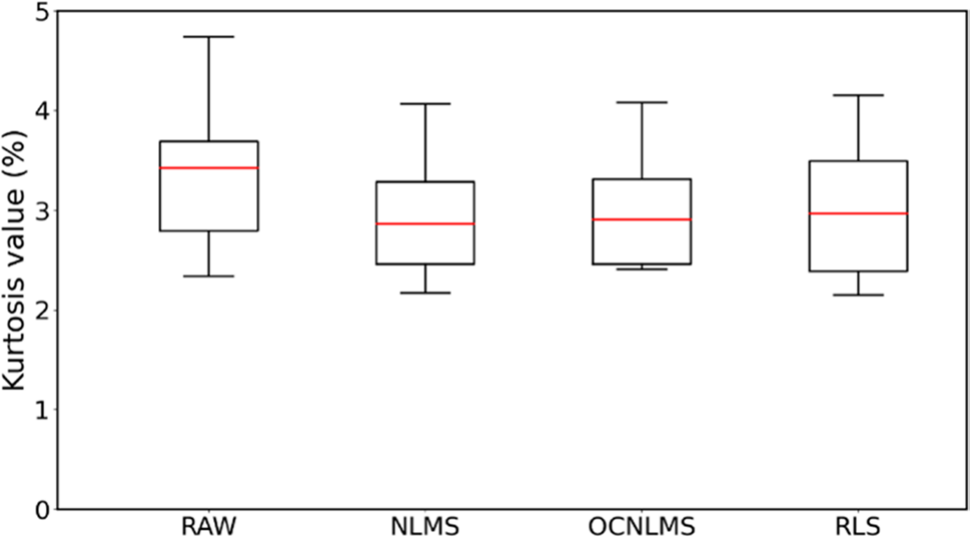
Comparison of kurtosis values between raw data (RAW) and data with different adaptive filters: NLMS, OCNLMS, and RLS. Results indicate that data with NLMS filter has the fewest outliers, as it has the lowest kurtosis value

The kurtosis values of resting HR for the eight sets of experiments, both for the raw data and the data after applying the NLMS, OCNLMS, and RLS filters, are shown in Table 4. Kurtosis is used to detect the presence of outliers in the data and provides an indication of the overall degree of outliers’ presence. A symmetric distribution is expected to have a kurtosis value of 3. The deviation of outliers from the normal distribution decreases as the kurtosis decreases [56]. According to the graph, the data processed using the NLMS filter exhibits the lowest kurtosis (2.98%), followed by the OCNLMS filter (3.01%) and the RLS filter (3.03%). All of the filters’ kurtosis levels are lower than that of the raw data (3.27%), as seen in Fig. 12, demonstrating their effectiveness in eliminating outliers of HR values.

**Table 4:** Kurtosis values of resting HR for raw data and data with different adaptive filters

Kurtosis value (%)	RAW	NLMS	OCNLMS	RLS
NLR 0.5	2.832	2.878	2.916	3.044
NLR 1	3.535	4.067	4.086	4.154
NUD 0.5	4.739	3.057	3.058	3.351
NUD 1	4.17	3.966	4.081	3.93
SLR 0.5	2.698	2.489	2.462	2.41
SLR 1	3.432	2.374	2.452	2.319
SUD 0.5	3.425	2.848	2.894	2.894
SUD 1	2.34	2.175	2.413	2.152
Mean	3.271375	2.98175	3.0115	3.03175

The experimental setup, with subject sitting still and employing only arm-driven movement, was designed to maintain a stable HR trend. In this context, smaller differences between HR estimates were preferable, as indicated by the mean of the differences between HR values when comparing raw data to data processed using NLMS, OCNLMS, and RLS filters, as presented in Table 5. The results indicate that the RLS filter exhibited the smallest difference of 1.6257 bpm, followed closely by NLMS with a difference of 1.628 bpm, and then OCNLMS with a difference of 1.6388 bpm. All of the filters’ mean difference are smaller than the raw data (1.7042 bpm). Considering the presence of more outliers in the OCNLMS results, it is reasonable to conclude that the NLMS filter outperforms the other two filters in accurately estimating resting HR in this specific experimental scenario.

**Table 5:** Comparison of mean difference between resting HR

Mean of difference between resting HR (bpm)	RAW	NLMS	OCNLMS	RLS
NLR0.5	1.7474	1.6374	1.6612	1.6376
NLR1	1.7972	1.7339	1.8708	1.7706
SLR0.5	1.6562	1.5123	1.5636	1.5691
SLR1	1.5324	1.6395	1.5322	1.5681
NUD0.5	2.1554	2.0053	1.905	1.9745
NUD1	1.7188	1.7792	1.7583	1.6898
SUD0.5	1.7139	1.4478	1.5546	1.484
SUD1	1.3124	1.2688	1.2647	1.3117
Mean	1.7042	1.628	1.6388	1.6257

Moreover, considering the relatively minor variations between the raw data and the filters in resting HR estimation, a comparison of the mean differences in instantaneous HR, as presented in Table 6, was undertaken. The influence of MA tends to have a more substantial impact on the estimation of instantaneous HR, resulting in larger mean difference values compared to resting HR. The results indicate that the NLMS filter yielded the smallest difference, measuring 7.8489 bpm, closely followed by RLS, which displayed a difference of 7.9582 bpm, and then OCNLMS, which exhibited a difference of 8.0058 bpm. Likewise, all of these filter-based mean differences proved to be smaller than that of the raw data, which showcased a mean difference of 10.6071 bpm.

**Table 6:** Comparison of mean difference between instantaneous HR

Mean of difference between Instantaneous HR (bpm)	RAW	NLMS	OCNLMS	RLS
NLR0.5	12.8716	8.6703	9.1568	9.0911
NLR1	9.3442	7.9694	7.9031	8.1363
SLR0.5	13.0859	8.6368	8.9876	8.7172
SLR1	8.7235	6.0588	6.5123	6.1998
NUD0.5	11.4548	8.6011	8.9916	8.852
NUD1	7.7831	7.2354	6.8796	7.1446
SUD0.5	12.3171	7.7666	8.1692	8.0047
SUD1	9.2765	7.8533	7.446	7.52
Mean	10.6071	7.8489	8.0058	7.9582

Indeed, it can be succinctly stated that NLMS consistently performs marginally better than the other adaptive filters, given the minimal variation in kurtosis values and mean difference HR values. Furthermore, it is evident that the adaptive filter excels in instantaneous HR estimation compared to resting HR, as indicated by the significant reduction in mean difference values (decreased from 10 to 8 bpm) following the application of the adaptive filter.

## Conclusion

In this paper, we present a non-invasive and continuous experimental set-up designed for monitoring SpO_2_ and HR to assess sleep apnea at home. Various breathing experiments were performed to investigate the relationship between SpO_2_, HR, and apnea. We also implemented various adaptive filters to compare their effectiveness in removing MA.

Several key findings have emerged from this study. In terms of SpO_2_, both customized and empirical formulas were employed for comparison. The data indicates that the bespoke formula yielded more accurate results than the empirical formula for calculating SpO_2_, with an error rate of 0% in SpO_2_*>*100% compared to 18.18% of empirical formula. In addition to this, we found up-down finger motion introduced more MA than left-right motion, and the errors in SpO_2_ estimation are increased as the frequency of movement increased. A delay between the onset of apnea and the subsequent fall in SpO_2_ levels has been found only in limited experiments because of MA. The comparison of the percentage of data within the whiskers reveals that both the OCNLMS and RLS filters have a slightly larger percentage of data within the whiskers when compared to the raw data, which itself had a percentage of 87.89%. Specifically, the OCNLMS filter achieved a percentage of 87.92%, while the RLS filter reached 88.46%. In contrast, the NLMS filter exhibited a slightly smaller percentage of data within the whiskers, recording a percentage of 87.43%. The differences between the various filter types and the raw data are relatively small, indicating that the filters have a subtle effect on the removal of motion artifacts. The inserting of adaptive filters during SpO_2_ estimation increased noise in certain cases rather than removing MA. The adaptive filter’s effectiveness in mitigating motion artifacts appears to be limited, likely due to the relatively low sampling frequency of 25 Hz for the SpO_2_ signal.

Regarding HR, MA have a greater impact on the instantaneous HR than the resting HR and a prolonged pause in breathing leads to an increase in HR to obtain more oxygen, followed by a gradually decreases and stabilization upon resumption of breathing. Our study also found that the NLMS filter have the lowest kurtosis value (2.98 bpm) among the compared filters. Additionally, the NLMS filter exhibits slightly superior performance in terms of mean differences for both resting HR (1.628 bpm) and instantaneous HR (7.8489 bpm) compared to other adaptive filters. Nevertheless, considering the modest nature of these variations, our conclusion is that NLMS consistently demonstrates a marginal advantage over the other adaptive filters. Moreover, the adaptive filter exhibits superior performance in instantaneous HR estimation compared to resting HR, as demonstrated by the noteworthy MA reduction in mean difference values (reduced from 10 to 8 bpm) following the application of the adaptive filter.

### Study limitations

Only a researcher herself is permitted to conduct the experiment by the university’s ethic committee, which may limit the generalization of the findings.

### Supplementary information

Below is the link to the electronic supplementary material.

Supplementary file1 (DOCX 1241 KB)
