# Supplementary material for: A comparative study of accuracy in major adaptive filters for motion artifact removal in sleep apnea tests
Source: Med Biol Eng Comput. 2023 Dec 5;62(3):829–42. doi: 10.1007/s11517-023-02979-9 (PMC10881614; doi:10.1007/s11517-023-02979-9)
Supplement: Supplementary file 1 — Supplementary file1 (DOCX 1241 KB) [file 11517_2023_2979_MOESM1_ESM.docx]

**Supplementary**

A Comparative Study of Accuracy in Major Adaptive Filters for Motion Artefact Removal in Sleep Apnoea Tests

*Yongrui Chen, Yurui Zheng, Sam Johnson, Richard Wiffen and Bin Yang*

Supplementary Fig. 1. Captured Normalized PPG signal. Red line represents red light signal, green line represents inferred signal.

Supplementary Fig. 2. Comparison of raw data vs data processed with NLMS, OCNLMS and RLS adaptive filters on raw PPG red and inferred signal. a. Raw red light signal b. Raw red-light signal filtered with NLMS filter c. Raw red-light signal filtered with OCNLMS filter d. Raw red-light signal filtered with RLS filter e. Raw inferred-light signal f. Raw inferred-light signal filtered with NLMS filter g. Raw inferred-light signal filtered with OCNLMS filter h. Raw inferred-light signal filtered with RLS filter.

Supplementary Fig. 3. Comparison of raw data vs data processed with NLMS, OCNLMS, RLS, LMS, AP, LLNCOSH and SSLMS adaptive filters on blood oxygen saturation during snore experiment with 1Hz up & down movement. a. RAW vs NLMS b. RAW vs OCNLMS c. RAW vs RLS d. RAW vs LMS e. RAW vs AP f. RAW vs LLNCOSH g. RAW vs SSLMS Note ‘F’ in the figure represents ‘freeze’ and indicates a stationary hand. ‘U&D’ represents up & down movement.

Supplementary Table I. Error rates for raw data and data processed with different adaptive filters.

Supplementary Fig. 4. Comparison of the error rates between raw data and data processed with different adaptive filters for SpO_2_ < 90% throughout the experiment: RAW vs. NLMS vs. OCNLMS vs. RLS vs. LMS vs. AP vs. LLNCOSH vs. SSLMS. Results indicate data with OCNLMS filter has fewer errors than other adaptive filters.

Supplementary Table II. Kurtosis values for raw data and data processed with different adaptive filters

Supplementary Fig. 5. Histogram of Resting HR of Raw data and data with NLMS, OCNLMS, RLS, LMS, AP, LLNCOSH and SSLMS filters on Snore Experiment with 0.5Hz Up & Down movement. a. RAW vs. NLMS vs. OCNLMS vs. RLS vs. LMS vs. AP vs. LLNCOSH vs. SSLMS b. RAW c. NLMS d. OCNLMS e. RLS f. LMS g. AP h. LLNCOSH i. SSLMS

Supplementary Fig. 6. Comparison of Kurtosis values for raw data and data with different Adaptive Filters: NLMS, OCNLMS, RLS, LMS, AP, LLNCOSH and SSLMS filters. Results indicate data with NLMS filter has the fewest outliers, as it has the lowest kurtosis value.


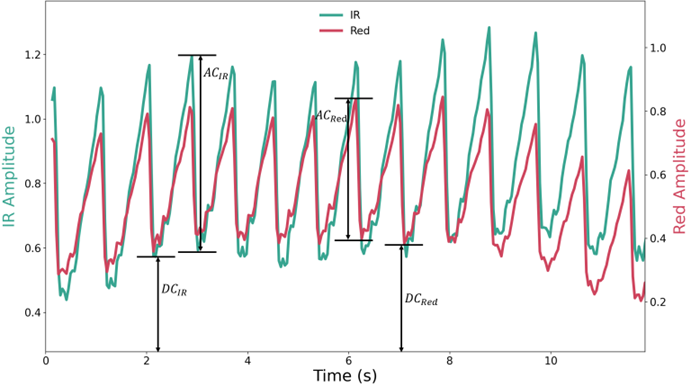


Supplementary Fig. 1. Captured Normalized PPG signal. Red line represents red light signal, green line represents infrared signal.


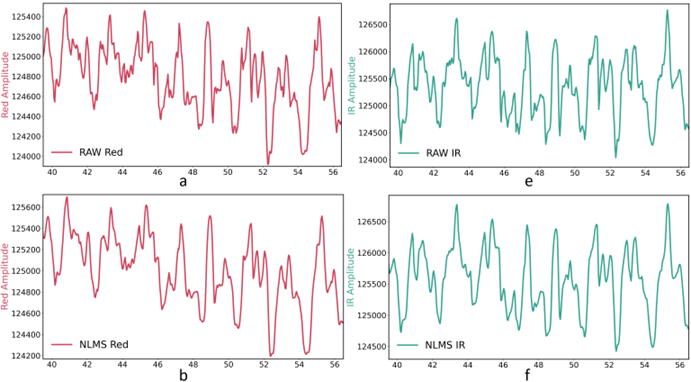


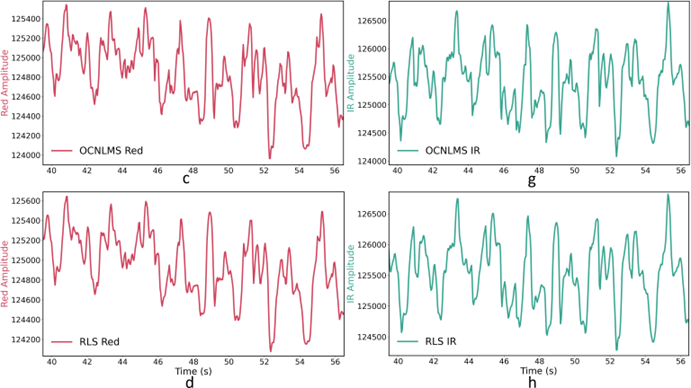


Supplementary Figure 2. Comparison of raw data vs data processed with NLMS, OCNLMS and RLS adaptive filters on raw PPG red and infrared signal. a. Raw red light signal b. Raw red-light signal filtered with NLMS filter c. Raw red-light signal filtered with OCNLMS filter d. Raw red-light signal filtered with RLS filter e. Raw infrared-light signal f. Raw infrared-light signal filtered with NLMS filter g. Raw infrared-light signal filtered with OCNLMS filter h. Raw infrared-light signal filtered with RLS filter.

The difference between the raw and filtered data of the red and infrared optical signals is limited, suggesting that the filter smooths out a portion of the data but doesn't eliminate abnormal fluctuations in the waveform.


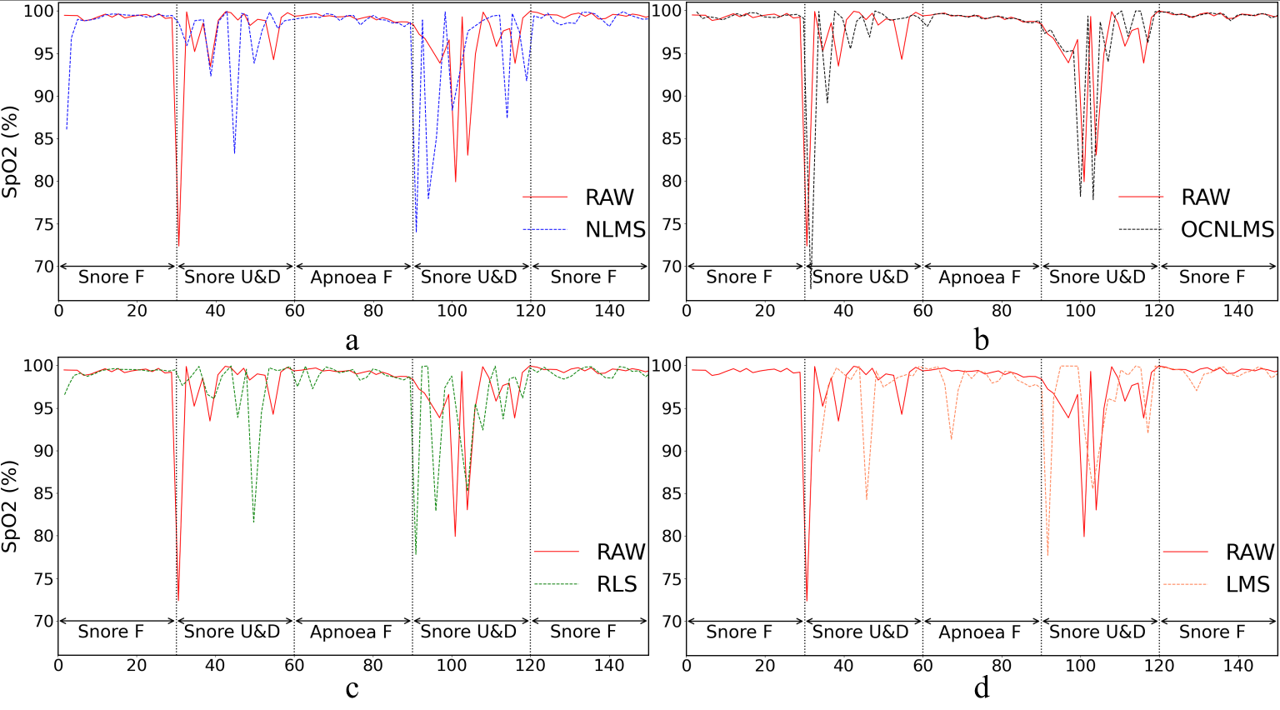


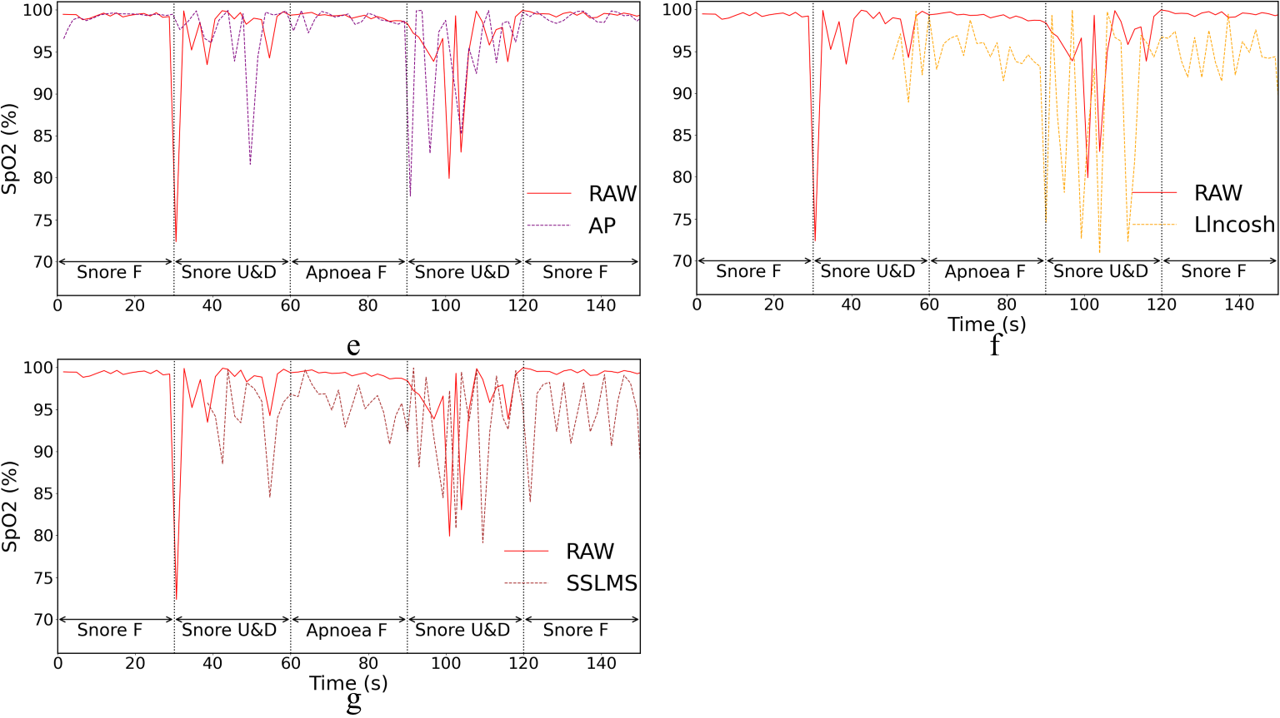


Supplementary Fig. 3. Comparison of raw data vs data processed with NLMS, OCNLMS, RLS, LMS, AP, LLNCOSH and SSLMS adaptive filters on blood oxygen saturation during snore experiment with 1Hz up & down movement. a. RAW vs NLMS b. RAW vs OCNLMS c. RAW vs RLS d. RAW vs LMS e. RAW vs AP f. RAW vs LLNCOSH g. RAW vs SSLMS Note ‘F’ in the figure represents ‘freeze’ and indicates a stationary hand. ‘U&D’ represents up & down movement.

The addition of the adaptive filters increases the noise in the data rather than removing the MA.

Supplementary TABLE I Error rates for raw data and data processed with different adaptive filters

| Error Rate (%) | SpO_2_ < 90% **RAW** | SpO_2_ < 90% **NLMS** | SpO_2_ < 90% **OCNLMS** | SpO_2_ < 90% **RLS** | SpO_2_ < 90% **LMS** | SpO_2_ < 90% **AP** | SpO_2_ < 90% **LLNCOSH** | SpO_2_ < 90% **SSLMS** |
| --- | --- | --- | --- | --- | --- | --- | --- | --- |
| NLR0.5 | 1.93 | 2.64 | 1.84 | 2.61 | 8.53 | 2.61 | 26.48 | 21.67 |
| NLR1 | 2.12 | 3.83 | 2.14 | 3.06 | 10.74 | 3.06 | 27.86 | 15.44 |
| NUD0.5 | 2.66 | 4.26 | 3.86 | 4.19 | 7.02 | 4.19 | 22.38 | 11.93 |
| NUD1 | 2.83 | 5.95 | 3.41 | 3.63 | 7.12 | 3.63 | 22.7 | 10.23 |
| SLR0.5 | 1.74 | 3.91 | 2.2 | 3.65 | 9.36 | 3.65 | 25.97 | 17.78 |
| SLR1 | 1.19 | 1.97 | 1.74 | 1.75 | 6.45 | 1.75 | 28.81 | 13.38 |
| SUD0.5 | 2.4 | 3.27 | 2.63 | 2.3 | 8.83 | 2.3 | 24.27 | 14.65 |
| SUD1 | 3.46 | 4.15 | 4.11 | 3.98 | 8.82 | 3.95 | 19.86 | 11.09 |
| **Mean** | **2.28** | **3.74** | **2.72** | **3.14** | **8.39** | **3.14** | **24.86** | **14.56** |


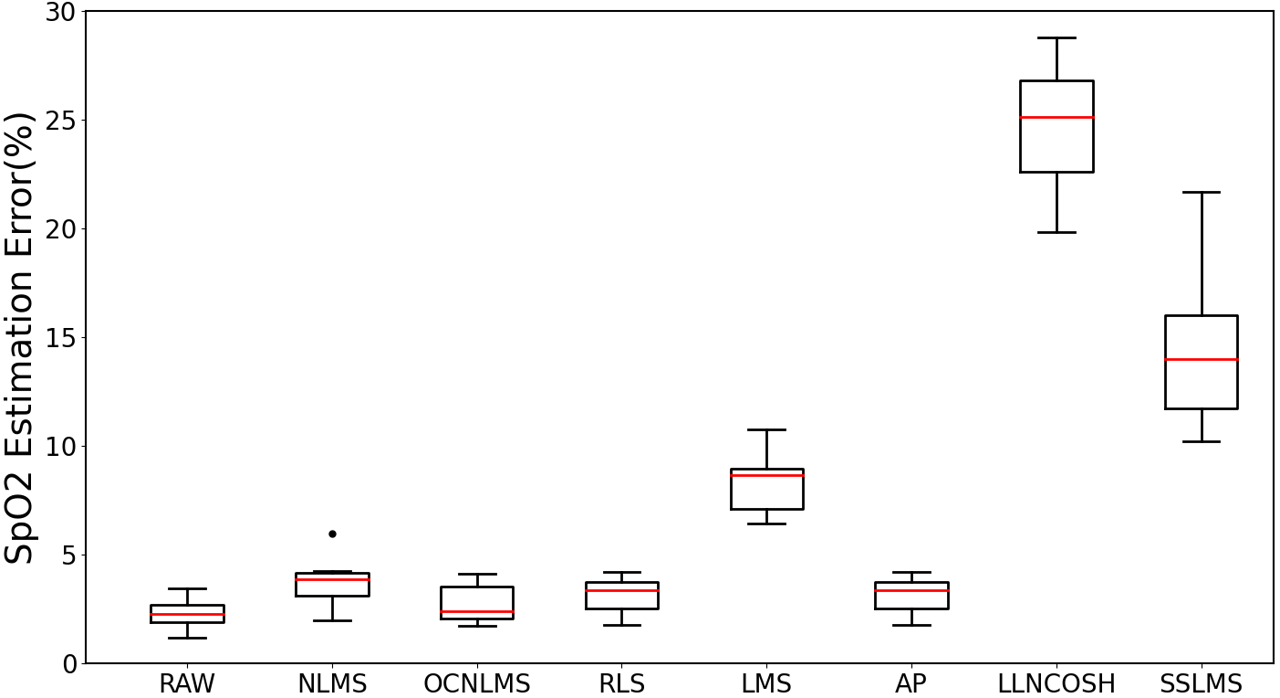


Supplementary Fig. 4. Comparison of the error rates between raw data and data processed with different adaptive filters for SpO2 < 90% throughout the experiment: RAW vs. NLMS vs. OCNLMS vs. RLS vs. LMS vs. AP vs. LLNCOSH vs. SSLMS. Results indicate data with OCNLMS filter has fewer errors than other adaptive filters.

Regarding the total error rate, the raw data without the adaptive filter exhibited the lowest error rate at 2.28% for SpO_2_*<*90% throughout the experiment, as depicted in the box plots. Comparing the data with the filters, the OCNLMS filter had the best result (2.72%), followed by the RLS and AP filters (3.14%) and the NLMS filter (3.74%), then the LMS filter (8.39%), the SSLMS filter (14.56%), while the LLCOSH filter had the largest error rate at 24.86%.


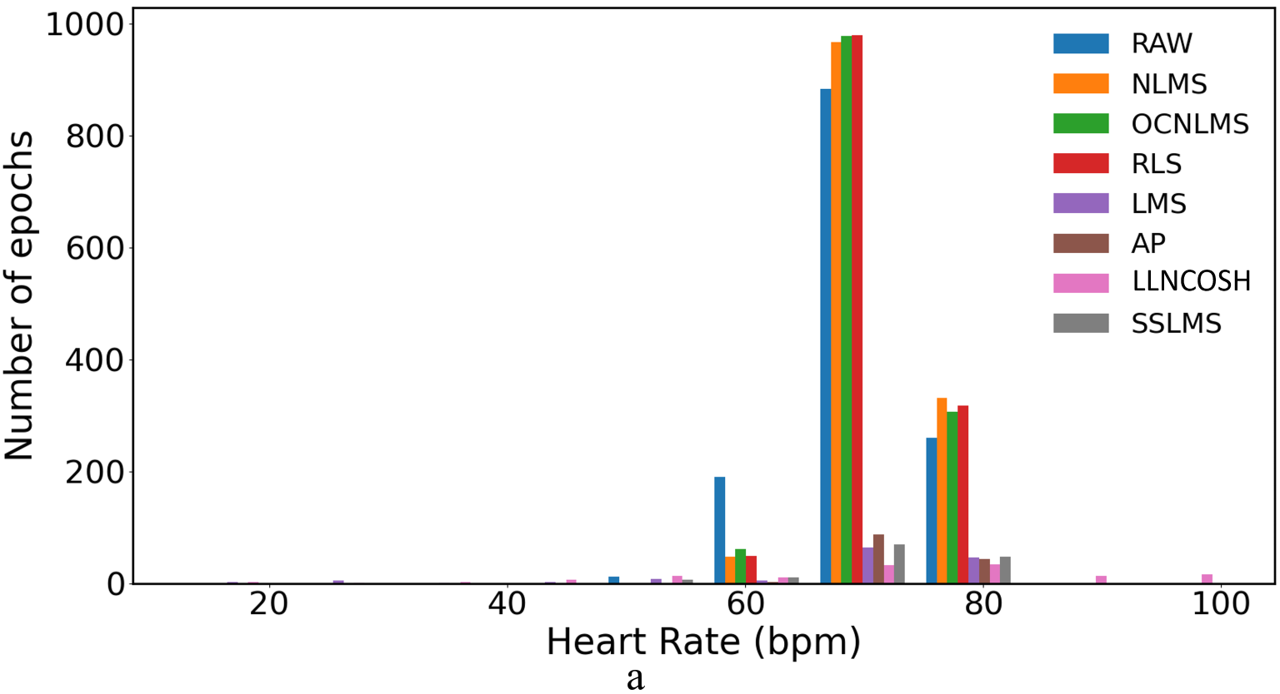

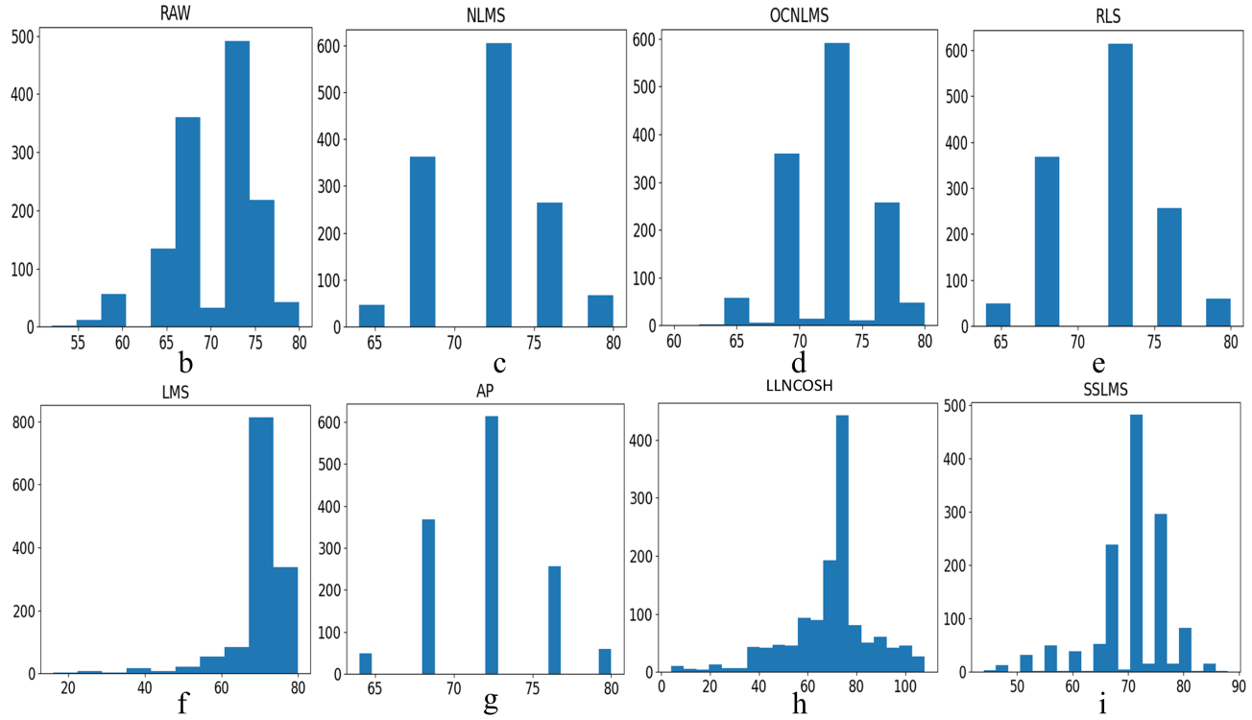


Supplementary Fig. 5. Histogram of Resting HR of Raw data and data with NLMS, OCNLMS, RLS, LMS, AP, LLNCOSH and SSLMS filters on Snore Experiment with 0.5Hz Up & Down movement. a. RAW vs. NLMS vs. OCNLMS vs. RLS vs. LMS vs. AP vs. LLNCOSH vs. SSLMS b. RAW c. NLMS d. OCNLMS e. RLS f. LMS g. AP h. LLNCOSH i. SSLMS

The graph depicts the distribution of the HR of the researcher during the experiment, with the HR mainly concentrated in the 60-80 bpm range. However, there are some obvious outliers in the data with the addition of the LMS, LLNCOSH and SSLMS filters.

Supplementary TABLE II Kurtosis values for raw data and data processed with different

adaptive filters

| Kurtosis value (%) | **RAW** | **NLMS** | **OCNLMS** | **RLS** | **LMS** | **AP** | **LLCOSH** | **SSLMS** |
| --- | --- | --- | --- | --- | --- | --- | --- | --- |
| NLR 0.5 | 2.832 | 2.878 | 2.916 | 3.044 | 11.039 | 3.044 | 5.102 | 3.654 |
| NLR 1 | 3.535 | 4.067 | 4.086 | 4.154 | 10.228 | 4.159 | 7.666 | 3.414 |
| NUD0.5 | 4.739 | 3.057 | 3.058 | 3.351 | 12.32 | 3.351 | 4.483 | 4.071 |
| NUD 1 | 4.17 | 3.966 | 4.081 | 3.93 | 11.972 | 3.93 | 4.935 | 3.476 |
| SLR 0.5 | 2.698 | 2.489 | 2.462 | 2.41 | 3.14 | 2.41 | 5.502 | 3.919 |
| SLR 1 | 3.432 | 2.374 | 2.452 | 2.319 | 5.468 | 2.319 | 4.837 | 2.452 |
| SUD 0.5 | 3.425 | 2.848 | 2.894 | 2.894 | 14.359 | 2.894 | 4.555 | 5.079 |
| SUD 1 | 2.34 | 2.175 | 2.413 | 2.152 | 7.795 | 2.152 | 4.571 | 2.768 |
| **Mean** | **3.2714** | **2.9818** | **3.0115** | **3.0318** | **9.5401** | **3.0324** | **5.2064** | **3.6041** |


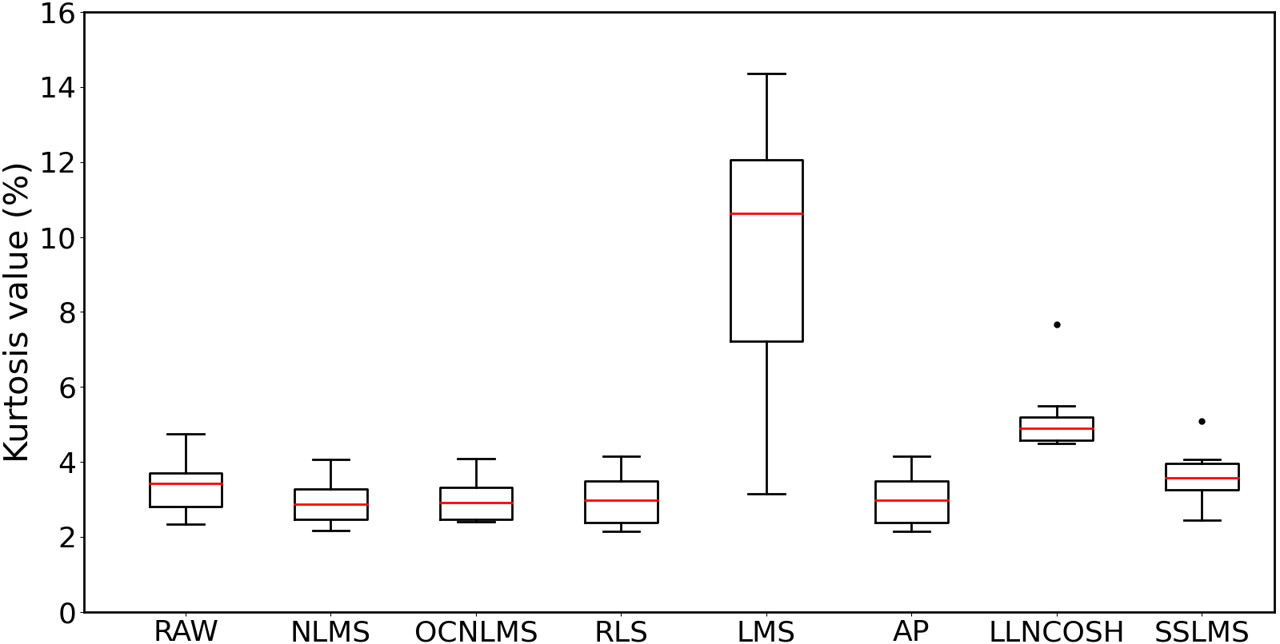


Supplementary Fig. 6. Comparison of Kurtosis values for raw data and data with different Adaptive Filters: NLMS, OCNLMS, RLS, LMS, AP, LLNCOSH and SSLMS filters. Results indicate data with NLMS filter has the fewest outliers, as it has the lowest kurtosis value.

According to the graph, the data processed using the NLMS filter exhibits the lowest kurtosis (2.98), followed by OCNLMS (3.01), RLS (3.0318) and AP (3.0324) filter. There are even more outliers observed with the addition of the SSLMS, LLCOSH and LMS filters, especially the LMS filter, which resulted in a kurtosis value of 9.54.
